# Brain structure correlates of foreign language learning experiences

**DOI:** 10.3389/fnhum.2025.1663218

**Published:** 2025-09-18

**Authors:** Xiaoqiao Wang, Jae-Yoon Kim, Jun-Ho Kim, Yoonseok Choi, Eun-Gyu Ha, Dong-Hyun Kim, Sunhae Sul, Sujin Yang, Hyun-joo Song

**Affiliations:** ^1^Department of Psychology, Yonsei University, Seoul, Republic of Korea; ^2^Department of Electrical and Electronic Engineering, Yonsei University, Seoul, Republic of Korea; ^3^Department of Psychology, Pusan National University, Busan, Republic of Korea; ^4^Department of Psychology, Ewha Womans University, Seoul, Republic of Korea

**Keywords:** foreign language learning, neuroimaging, gray matter volume, white matter integrity, structural MRI

## Abstract

Previous research demonstrates that bilingual experiences produce dynamic and variable structural adaptations in the brain. However, most studies focus on individuals who naturally acquire multiple languages, leaving the neural effects of long-term foreign language learners with limited exposure less understood. In this study, we examined 32 young adult long-term foreign language learners in South Korea to determine how age of acquisition, proficiency, and daily use shape gray matter volume and white matter integrity in predominantly monolingual environments. We found structural changes in brain regions involved in language control and executive function. Voxel-based morphometry analysis revealed that earlier foreign language acquisition was significantly associated with greater gray matter volume in the left inferior parietal lobule (uncorrected *p* < 0.001) and the left thalamus (uncorrected *p* < 0.001). Higher daily use was significantly associated with greater volume in the right inferior parietal lobule (uncorrected *p* = 0.001) but reduced it in the right anterior cingulate cortex (uncorrected *p* < 0.001). Tract-based spatial statistics analysis further showed that earlier age of acquisition was significantly associated with better white matter integrity in the splenium of the corpus callosum (FWE-corrected *p* = 0.009), while higher daily use was significantly associated with better integrity in the body of the corpus callosum (FWE-corrected *p* = 0.030). These findings suggest that even limited but sustained exposure to a foreign language can lead to significant structural adaptations, broadening our understanding of language-related neuroplasticity beyond traditional bilingual contexts.

## 1 Introduction

Using more than one language shapes brain structure, a phenomenon widely observed in bilingual groups (Abutalebi et al., 2015a; DeLuca et al., 2019, 2020; Grundy et al., 2017; Luk et al., 2011; Marin-Marin et al., 2022; Mechelli et al., 2004; Olulade et al., 2016; Pliatsikas et al., 2015). Research consistently shows that bilingual groups develop greater gray matter volume than monolinguals in regions such as the anterior cingulate cortex (ACC), inferior parietal lobule (IPL), inferior frontal gyrus (IFG), and basal ganglia (Abutalebi et al., 2015a,b; Burgaleta et al., 2016; DeLuca et al., 2024; Mechelli et al., 2004). These areas play central roles in language control and executive functions (Green and Abutalebi, 2013; Grundy et al., 2017).

Researchers have also reported similar bilingualism-related differences in white matter tracts. Aged and young bilingual groups show better white matter integrity than their monolingual counterparts in tracts such as the bilateral superior longitudinal fasciculus (SLF), the inferior fronto-occipital fasciculus (IFOF), and the corpus callosum (Luk et al., 2011; Pliatsikas et al., 2015). These structural adaptations—including increased gray matter volume in executive control regions and enhanced white matter integrity—appear to support cognitive advantages often observed in bilinguals, such as improved attentional control, task switching, and working memory (Alrwaita et al., 2024; Bialystok, 2011; DeLuca et al., 2020; Green and Abutalebi, 2013; Miyake et al., 2000).

Although bilingualism promotes structural adaptations, these changes vary across learners’ backgrounds and language experiences. For instance, while bilingualism generally relates to extended cortical volume, evidence suggests that this relationship changes with the stage of second language (L2) immersion (Grundy et al., 2017; Pliatsikas et al., 2020). Simultaneous interpreters, who are typically bilinguals at later stages of L2 immersion, demonstrate decreased volume in regions such as the left ACC, bilateral IFG, and left insula, changes that correlate with cumulative hours of interpreting (Elmer et al., 2014). Moreover, DeLuca et al. (2020) proposed that specific experience-based bilingual factors uniquely shape brain structure. They found that the intensity and diversity of language use influence cortical changes in regions supporting language processing and executive control, whereas the duration of L2 usage promotes neural efficiency, reflected in changes to subcortical structures and white matter pathways (DeLuca et al., 2024). These findings highlight the need to move beyond treating bilinguals as a homogeneous group and examine how specific aspects of language experience such as language proficiency, age of acquisition (AoA), and daily use frequency, drive neural adaptation (e.g., Bialystok et al., 2012; DeLuca et al., 2020; Luk and Bialystok, 2013).

Despite these advances, most neuroimaging studies focus on traditional bilinguals who naturally acquire two (or more) languages and use them regularly in interactional contexts. Far less is known about groups with non-immersive dual-language experiences, those who neither learn a second language naturally nor are immersed in a bilingual environment. This study addresses that gap by focusing on a large but understudied group: long-term foreign language learners with limited daily exposure to the target language. These learners typically study their foreign language—English—in formal educational settings for at least 12 years, but rarely use it in everyday life. As a result, they often achieve high proficiency but accumulate little practical experience. Such profiles are especially common in East Asia, including China and South Korea, where English education is extensive but real-world exposure to English outside the classroom remains minimal. Despite the growth of bilingualism research, scholars have largely overlooked how this distinct type of language experience relates to neural adaptation.

This foreign language learner population differs from traditional bilinguals and most studied second language learners in two significant ways. First, the context and goals of their learning differ. Their language learning occurs primarily in academic settings and also arises from achievement-oriented goals rather than organic exposure (Shin et al., 2018). Since they do not live in multilingual environments, they lack opportunities for spontaneous, real-time oral interaction in the target language. It remains unclear whether such differences in language experience lead to different brain restructuring. Second, East Asian learners typically study a foreign language, mostly English, over an extended period. Previous neuroimaging studies on L2 learning have mostly examined short-term training effects (e.g., Legault et al., 2019; Mårtensson et al., 2012). Whether long-term foreign language learning produces comparable or distinct neural effects remains an open question.

Converging evidence suggests that even limited exposure to a foreign language can influence cognitive and neural development. Infants exposed to a non-native language can discriminate phonetic features absent in their native tongue (Kuhl et al., 2003). Young monolingual children with minimal Spanish exposure successfully acquired novel words from Spanish speakers (Akhtar et al., 2012). Children with limited non-native language input have also shown greater openness to dual labeling (Rojo and Echols, 2018), particularly in socially interactive contexts (Lee and Song, 2024). Among adults, short-term L2 training enhances cognitive performance (Wong et al., 2019), strengthens functional connectivity (Bubbico et al., 2019), and even induces structural brain changes (Mårtensson et al., 2012). Together, these findings suggest that, even without immersive environments, long-term foreign language learning may yield measurable neuroplastic changes.

Building on this work, the present study investigates how gray matter volume and white matter integrity are associated with age of acquisition, foreign language proficiency, and daily use experiences among long-term English learners in South Korea, a predominantly monolingual environment. The participants in this study began learning English since childhood, primarily through formal instruction with minimal opportunities for everyday communicative use, giving them a distinct profile from those in previous studies. We hypothesize that, despite their restricted daily exposure, they will exhibit structural adaptations linked to language-related factors in gray matter volume and white matter integrity in regions involved in language and cognitive control, following patterns observed in traditional bilinguals.

## 2 Materials and methods

### 2.1 Participants

Thirty-two healthy, right-handed young adults participated in the study (19 females; age range: 19–31 years, M = 23.66, SD = 2.68). We used G*Power to calculate the required sample size for a multiple regression with six predictors (power = 0.80, α = 0.05), based on an effect size (f^2^ = 0.276) estimated from the correlation reported in Abutalebi et al. (2015b), a study closely related to ours. All participants were native Korean speakers who began learning English as their first foreign language during childhood (AoA: M = 6.47, SD = 2.81). None had lived abroad before adulthood, and all reported less than two years of overseas experience afterward. Table 1 presents the demographic characteristics of their backgrounds. The Institutional Review Board of Yonsei University approved all recruitment and study procedures. We obtained informed consent from every participant prior to participation.

**TABLE 1:** Demographic and language learning background of participants (*N* = 32).

Variable	Value
Age (years), M (SD)	23.66 (2.68)
Gender (female/male)	19/13
Educational background	24 undergraduate students; 8 graduate students
Years of English study, M (SD)	17.23 (3.67)
Prior immersion experience	6 with > 6 months abroad; 1 with > 3 months abroad; 25 none

### 2.2 Materials and procedures

#### 2.2.1 Language and social background questionnaire

All participants first completed the Language and Social Background Questionnaire (LSBQ, Anderson et al., 2018), which assesses social background, language history, and language use experiences across different contexts. The LSBQ is widely used in bilingualism studies, and its self-rated proficiency and frequency items have demonstrated good reliability and validity (Anderson et al., 2018; DeLuca et al., 2019). We did not use the original composite bilingualism score, which better suits traditional bilinguals whose home and social language experiences differ. Instead, we calculated three separate scores from the LSBQ: AoA, English proficiency, and daily English use. These predictors serve as independent variables of interest because they capture distinct aspects of individual language-related experience and are widely considered in bilingualism research (e.g., DeLuca et al., 2019, 2020). AoA reflects the developmental timing of foreign language acquisition. Proficiency indicates achieved language competence, while daily use reflects the ongoing cognitive demands of language switching and management.

Participants self-reported their AoA directly in the questionnaire. We assessed English proficiency using item 17.1 of the LSBQ, which asked participants to rate their speaking, comprehension, reading, and writing abilities on a scale from 0 (no proficiency) to 10 (high proficiency). We summed the four ratings to produce a composite proficiency score ranging from 0 to 40. We measured daily English use with 20 sub-items from LSBQ items 20–22. Items 20 and 21 asked participants to rate their English use across diverse contexts (e.g., school, home, with friends, religious activities) and activities (e.g., reading, media consumption, singing, social media use), each on a scale from 0 (entirely in Korean) to 4 (entirely in English). Item 22 asked about the frequency of language switching within individual conversations, rated on a separate 0–4 scale (0 = *never*, 4 = *very often*). We summed the sub-item scores to produce a composite daily use score ranging from 0 to 80, with higher values reflecting greater use of English in everyday contexts.

#### 2.2.2 Magnetic resonance imaging (MRI) data acquisition

Neuroimaging data were acquired on a Siemens Vida 3T scanner at the Siemens Healthineers Research MRI Center, Yonsei University. Each participant underwent a T1 MPRAGE anatomical scan and a diffusion-weighted imaging scan. We acquired anatomical T1-weighted images with a three-dimensional MPRAGE sequence of 208 contiguous 1 mm-thick axial slices (repetition time (TR) = 2,200 ms; echo time (TE) = 2.91 ms; flip angle = 8°; field of view (FOV) = 256 × 256 mm; voxel size = 1.0 × 1.0 × 1.0 mm; acceleration factor = 2). We acquired diffusion-weighted images using echo planar imaging with 99 contiguous 1.5 mm-thick axial slices (TR = 9,000 ms; TE = 104 ms; *b*-value = 900 s/mm^2^; FOV = 240 × 240 mm; voxel size = 1.5 × 1.5 × 1.5 mm), applying diffusion gradients in six directions and a multiband acceleration factor of three. The total scan time was 5:07 min for T1 MRPRAGE and 9:49 min for DTI.

#### 2.2.3 MRI data preprocessing

For gray matter volume analysis, we preprocessed T1-weighted anatomical images using the Computational Anatomy Toolbox (CAT12; Gaser et al., 2024), an extension of Statistical Parametric Mapping (SPM12^1^), running on MATLAB R2023b (MathWorks Inc.). Following the standard CAT12 protocol, we first bias-corrected the images and segmented them into gray matter, white matter, and cerebrospinal fluid. We then performed an affine registration to a standard template, followed by high-dimensional DARTEL normalization to MNI space.

We assessed data quality through visual inspection and sample homogeneity checks provided by CAT12. We smoothed the resulting modulated and normalized gray matter segments within an 8 mm full-width at half-maximum (FWHM) Gaussian kernel to improve the signal-to-noise ratio and accommodate inter-individual anatomical variability. We also estimated total intracranial volume (TIV) during this preprocessing stage and later included it as a covariate in the statistical analyses to account for individual differences in overall brain size.

Diffusion-weighted images underwent standard preprocessing using the FMRIB Software Library’s Diffusion Toolbox (FSL; Jenkinson et al., 2012). First, we corrected susceptibility distortions with the *topup* pipeline and addressed motion-related signal outliers with the *eddy* tool. We then fitted a tensor model to the corrected data using *DTIFIT*, generating fractional anisotropy (FA) and mean diffusivity (MD) maps. FA values reflect the degree to which water diffusion is directionally constrained, indicating the alignment and coherence of white matter fiber (Beaulieu, 2002). MD measures the overall magnitude of water diffusion within tissue, providing insight into tissue density and cellular integrity (Alexander et al., 2007). In general, researchers interpret higher FA and lower MD values as indicators of greater white matter integrity. We visually inspected all processed images to ensure data quality. We then registered FA and MD images non-linearly to the FRMIB58_FA template in the MNI-152 standard space and skeletonized them using Tract-Based Spatial Statistics (TBSS; Smith et al., 2006) as implemented in FSL. We created a mean FA skeleton, thresholded at FA > 0.2 to include only major white matter tracts, and aligned MD images using the same transformation parameters before projecting them onto the mean FA skeleton for statistical analysis.

#### 2.2.4 MRI data and statistical analysis

Before the main neuroimaging analyses, we conducted descriptive and correlational analyses among the three questionnaire-derived predictors: AoA, English proficiency, and daily English use. We then performed voxel-wise multiple regression analyses using the general linear model (GLM) in SPM12 for gray matter volume, and the *randomise* tool in FSL for white matter integrity. In both modalities, we entered the three language-related predictors simultaneously as continuous variables of interest, while controlling for age and gender as covariates. For gray matter volume, we also included total intracranial volume (TIV) as a covariate to control for individual differences in head size.

We conducted voxel-based morphometry (VBM) analysis on the T1-weighted data using the general GLM in SPM12 to examine the relationship between gray matter volume and three language-related predictors collected from the questionnaire: AoA, proficiency, and daily use. We included age, gender, and TIV as covariates of no interest to reduce potential confounding effects. Analyses targeted cortical and subcortical gray matter structures, restricted to predefined regions of interest (ROIs) selected according to the UBET model (DeLuca et al., 2020, 2024) and previously associated with bilingualism (DeLuca et al., 2024; Elmer et al., 2014; Grundy et al., 2017). Cortical ROIs included the bilateral ACC, angular gyrus (part of IPL), IFG, and medial frontal gyrus (MFG). Subcortical ROIs included the bilateral caudate, putamen, and thalamus, as defined in the Neuromorphometrics atlas^2^.

We initially thresholded voxel-wise statistical maps at an uncorrected voxel-level threshold of *p* < 0.001. To compensate for multiple comparisons, we applied cluster-level family-wise error (FWE) correction using AFNI’s 3dClustSim tool^3^, which estimates cluster-size thresholds based on Monte Carlo simulations. We estimated the spatial autocorrelation function (ACF) from the GLM residuals and incorporated it into the simulation to determine the minimum cluster size corresponding to an FWE-corrected *p* < 0.05. Only clusters exceeding this threshold were considered significant and reported.

We assessed white matter integrity using TBSS in FSL, focusing on FA and MD values. For each metric, we performed voxel-wise GLM analyses with the *randomise* tool, running 5,000 permutations and applying threshold-free cluster enhancement (TFCE; Smith and Nichols, 2009) to correct for multiple comparisons. We again tested AoA, proficiency, and daily use as predictors while including age and gender as nuisance covariates. Analyses focused on predefined white matter tracts of interest (TOIs) identified in previous bilingualism research (DeLuca et al., 2024; Pliatsikas et al., 2015): the corpus callosum (CC; genu, body, and splenium), bilateral SLF, and IFOF, as defined in the JHU ICBM-DTI-81 White Matter Labels Atlas (Mori et al., 2008). We reported only clusters that survived a corrected threshold of *p* < 0.05 and contained more than 10 contiguous voxels.

## 3 Results

Table 2 presents descriptive statistics and intercorrelations for self-reported proficiency, AoA, and daily use of English. First, participants reported relatively high English proficiency (M = 25.73, SD = 5.71 out of 40). To supplement these self-reports, we collected certified English test scores, including the Test of English as a Foreign Language (TOEFL), Test of English for International Communication (TOEIC), commonly used in Asia and Europe, and Test of English Proficiency (TEPS), developed by Seoul National University in Korea. We converted all scores to TOEFL equivalents using the official conversion table: https://www.teps.or.kr/InfoBoard/ConversionTable#. The mean converted score reached 102.34 (SD = 9.81), substantially higher than the 2024 global average TOEFL score of 86 (ETS, 2025). Certified scores correlated strongly with self-reported proficiency (*r* = 0.70, *p* < 0.001), confirming the validity of the self-report measure.

**TABLE 2:** Descriptive statistics and correlations of participants’ language-related factors.

Variables	Mean (SD)	1. AoA	2. English proficiency
AoA	6.47 (2.81)	–	–
English proficiency	25.73 (5.71)	−0.55***	–
Daily English use	17.41 (9.52)	0.03	0.23

***P < 0.001.

Second, participants reported low daily English use (M = 17.41, SD = 9.52 out of 80). We derived this score from 20 LSBQ items that measured English use across diverse situations and activities, as well as language switching within conversations. The mean score represents less than one-quarter of the maximum possible, reflecting participants’ reliance on Korean in most personal and media contexts and their infrequent engagement in language switching. None of the participants had lived abroad before adulthood, and all reported fewer than 2 years of overseas experience afterward. Together, the low daily use scores from the LSBQ and the minimal residence abroad operationalize “limited exposure,” capturing participants’ scarce opportunities for naturalistic, immersive English use despite their relatively high proficiency.

Third, English proficiency correlated significantly with AoA (*r* = −0.64, *p* = 0.001), suggesting a link between earlier acquisition of English and higher proficiency. In contrast, neither AoA nor proficiency correlated significantly with daily English use (*r* = 0.03, *n.s.*; *r* = 0.23, *n.s.*, respectively).

**FIGURE 1:**
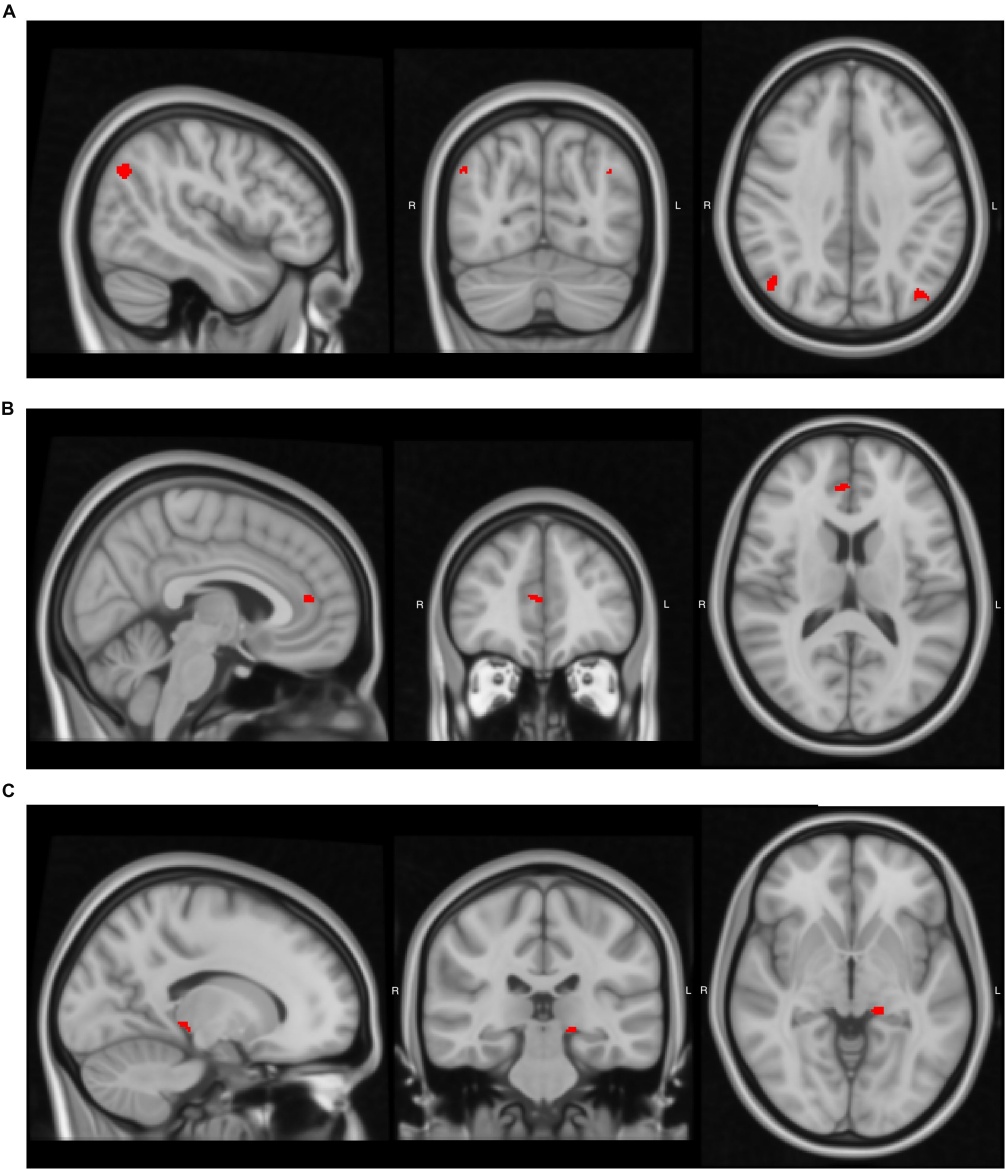
Brain regions where language-related factors significantly predicted gray matter volume. Detailed statistical values, cluster sizes and peak coordinates are reported in Table 3. **(A)** Age of acquisition (AoA) negatively predicted gray matter volume in the left angular gyrus, and daily use positively predicted volume in the right angular gyrus. **(B)** Daily use negatively predicted gray matter volume in the right anterior cingulate cortex (ACC). **(C)** AoA negatively predicted gray matter volume in the left thalamus. Images were generated using FSLeyes (FSL, RRID:SCR_002823).

After establishing the behavioral relationships among AoA, proficiency, and daily use, we examined whether these language-related experiences correlated with brain structure. Using VBM analysis, we tested whether gray matter volume in bilingualism-related regions varied with these language experiences (Table 3). Volume in the left angular gyrus increased with earlier AoA (uncorrected *p* < 0.001), while volume in the right angular gyrus increased with greater daily English use (uncorrected *p* = 0.001; Figure 1A). Daily English use also predicted reduced volume in the right ACC (uncorrected *p* < 0.001; Figure 1B). Among the subcortical structures, later AoA predicted reduced volume in the left thalamus (uncorrected *p* < 0.001; Figure 1C). Overall, earlier AoA correlated with greater gray matter volume in several regions, whereas daily English use predicted distinct patterns of structural adaptations. We did not observe other significant relationships. Supplementary Table 1 provides a complete list of significant and non-significant VBM results across the ROIs.

**TABLE 3:** Significant predictors of volume in cortical and subcortical regions of interest.

Language-related factors	Regions	Hemisphere	Direction	Peak *p*	*k*	*x*	*y*	*z*
AoA	Angular gyrus	Left	−	0.000	85	−40	−74	34
	Thalamus	Left	−	0.000	40	−15	−30	−6
Daily use	Anterior cingulate cortex	Right	−	0.001	42	4	42	12
	Angular gyrus	Right	+	0.000	84	46	−64	36

The reported *p*-values correspond to the uncorrected *p*-value of the peak voxel in each region. *k* refers to the cluster size, and the *x, y, z* coordinates represent the peak voxel location within each cluster in standard MNI (Montreal Neurological Institute) space.

**FIGURE 2:**
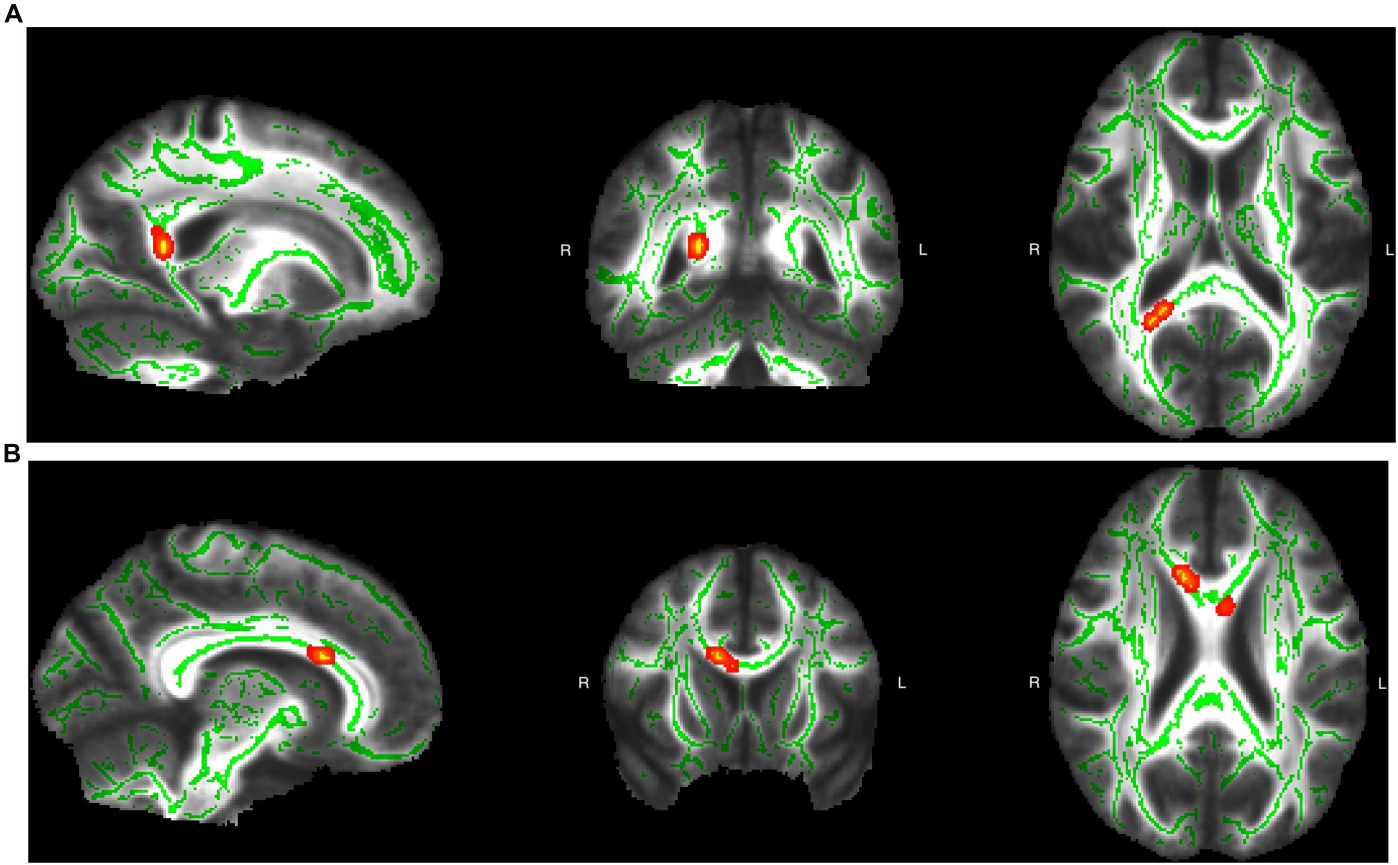
The corpus callosum (CC) tracts where language-related factors significantly predicted mean diffusivity (MD). We display significant clusters as filled areas to highlight the spatial extent of the effects. Table 4 reports the detailed statistical values, cluster sizes, and peak coordinates. **(A)** Age of acquisition (AoA) positively predicted integrity in the splenium of the CC. **(B)** Daily use negatively predicted integrity in the body of CC. Images were generated using FSLeyes (FSL, RRID:SCR_002823).

We then tested whether white matter integrity was associated with language experiences using TBSS analysis (Table 4). We found that AoA positively predicted MD in the splenium of the CC (FWE-corrected *p* = 0.009), whereas daily use negatively predicted MD in the body of the CC (FWE-corrected *p* = 0.030; Figure 2). We observed no significant effects for FA or in other TOIs. Supplementary Table 2 provides the complete list of significant and non-significant TBSS results.

**TABLE 4:** Significant predictors of white matter integrity in tracts of interest.

Language-related factors	Tracts	Measure	Direction	Peak *p*	*k*	*x*	*y*	*z*
AoA	Corpus callosum (splenium)	MD	+	0.015	31	24	−51	22
			+	0.009	27	20	−46	13
Daily use	Corpus callosum (body)	MD	−	0.030	40	7	15	20
			−	0.030	19	−8	3	26

The reported *p*-values correspond to the FWE-corrected *p*-value of the peak voxel in each region. *k* refers to the cluster size, and the *x, y, z* coordinates represent the peak voxel location within each cluster in standard MNI (Montreal Neurological Institute) space.

## 4 Discussion

The present study investigated whether long-term foreign language learning under conditions of limited daily exposure relates to structural brain adaptations. We focused on a unique and understudied group: long-term foreign language learners in South Korea, a linguistically homogeneous country where opportunities for foreign language use are scarce. These learners acquired English in formal educational settings over an extended period but had little exposure in everyday communication. Unlike traditional bilinguals frequently studied in previous research, who use their second language regularly in immersive environments, this group allows us to examine neuroplasticity that emerges under constrained experiential input. Prior studies show that even minimal exposure can shape language learning patterns (Bubbico et al., 2019; Kuhl et al., 2003; Lee and Song, 2024; Wong et al., 2019), leading us to hypothesize that sustained but limited experience can still induce meaningful neurocognitive changes. Accordingly, we examined how gray matter volume and white matter integrity in bilingualism-related regions varied with age of acquisition (AoA), English proficiency, and daily use.

Our VBM analyses revealed significant differences in the bilateral IPL, the right ACC, and the left thalamus. Specifically, earlier AoA (i.e., lower numerical AoA values) predicted greater gray matter volume in the left angular gyrus, a part of IPL known to play a central role in language learning and semantic processing (Barbeau et al., 2017; Van Ettinger-Veenstra et al., 2016). In our study, AoA correlated negatively with gray matter volume in this region, meaning that participants who learned English earlier had greater gray matter volumes. Daily use then positively predicted gray matter volume in the right angular gyrus. These findings align with previous research on bilingualism. For instance, Mechelli et al. (2004) found that early and late bilinguals exhibited increased gray matter density in the bilateral IPL and that L2 AoA correlated negatively with left IPL density. Similarly, Abutalebi et al. (2015b) reported that the left IPL correlated with L2 proficiency, whereas the right IPL linked to L2 exposure, results highly consistent with ours. Together, our findings replicate and extend prior work in bilinguals, showing that even long-term foreign language learning with limited exposure can reshape bilateral IPL structures involved in language processing.

We also found that higher daily English use predicted reduced gray matter volume in the right ACC, consistent with results from advanced bilinguals (e.g., Elmer et al., 2014). Grundy et al. (2017) proposed that highly proficient bilinguals exhibit reduced yet more efficient activation and decreased gray matter volume in the anterior regions, suggesting a neural optimization effect. The Dynamic Restructuring Model (Pliatsikas, 2020; Pliatsikas et al., 2020) similarly suggests that reductions in gray matter volume among proficient bilinguals may reflect increased efficiency in language and cognitive control mechanisms. Supporting these models, structural and functional studies show that bilinguals recruit the ACC more efficiently during cognitive tasks, with decreased activation linked to reduced volume, indicating improved language conflict management (Abutalebi et al., 2012). Therefore, our findings suggest that long-term foreign language learning, even with minimal daily exposure, strengthens cognitive control systems. An alternative interpretation is that reduced volume may reflect weakened control in this area; however, converging behavioral and neuroimaging evidence makes this less likely. Notably, Liu et al. (2021) found that learners with similar profiles to our participants exhibited decreased left ACC volume associated with better performance in language-switching tasks, reinforcing the interpretation that reduced ACC volume reflects functional efficiency. Although our results involved the right ACC, they likely reflect a similar optimization process.

In the subcortical regions, earlier AoA predicted greater gray matter volume in the left thalamus. This finding aligns with previous research (e.g., Burgaleta et al., 2016; Korenar et al., 2023), which identified the thalamus as a key relay structure supporting language and domain-general learning (Ayyildiz et al., 2025; Bulut and Hagoort, 2024; Grillner and Robertson, 2016). Researchers also report thalamic adaptations as a later stage of neuroplasticity, supporting more effective information relay and cognitive control across distributed networks (Green and Abutalebi, 2013; Grundy et al., 2017; Pliatsikas et al., 2020). Along with the observed reduction in right ACC volume, these findings together further suggest that long-term learning with limited or absent immersive L2 environments can trigger advanced stages of structural adaptation resembling those in experienced bilinguals.

Our diffusion-weighted imaging data revealed significant structural changes in white matter integrity. Specifically, earlier AoA and greater daily use predicted increased integrity in different parts of the CC, a major interhemispheric communication tract. Similar results have been widely reported in diverse bilingual populations (Luk et al., 2011; Pliatsikas et al., 2015), and even after short-term L2 training (Schlegel et al., 2012). Although the CC is not language-specific, it likely supports conflict management during language-switching, which may explain the observed structural differences. These results support our hypothesis that long-term L2 learning—even under minimal daily exposure—facilitates white matter reorganization that enhances efficiency in language and cognitive processing. However, diffusion metrics such as FA and MD provide only indirect indices of integrity, as multiple microstructural properties contribute to these metrics. Future studies should employ more biologically specific diffusion models to enhance interpretability.

Overall, our findings demonstrate that structural adaptations commonly observed in bilinguals also emerge in long-term foreign language learners with minimal exposure. Earlier AoA predicted increased gray matter volume in the left angular gyrus and left thalamus, along with enhanced white matter integrity in the splenium of CC. Greater daily use predicted increased gray matter volume in the right angular gyrus and enhanced integrity in the body of the CC, but reduced gray matter volume in the right ACC. Together, these patterns of combining increases, decreases, and white matter reorganization mirror the dynamic adaptations reported in bilingual populations.

We did not find any significant effects of the composite English proficiency score on gray matter regions or white matter tracts. One plausible explanation is that proficiency levels in our sample were relatively homogeneous, with most participants already achieving high academic proficiency, reducing variance. Another possibility is that the composite score averaged across speaking, comprehension, reading, and writing obscured skill-specific neural associations. To address this, we conducted an additional analysis entering each self-reported skill separately. Interestingly, we found that self-reported speaking proficiency correlated positively with gray matter volume in the left pars triangularis, a part of IFG (cluster size = 108 voxels, peak uncorrected *p* = 0.000, MNI coordinates *x* = −52, *y* = 34, *z* = 12). This region is a key part of Broca’s and is well-known to be involved in speech production and bilingualism (Green and Abutalebi, 2013; Indefrey and Levelt, 2004). Writing proficiency correlated positively with FA value in the body of the CC (cluster size = 22 voxels, peak uncorrected *p* = 0.001, FWE-corrected *p* = 0.034, MNI coordinates *x* = −9, *y* = −1, *z* = 28), suggesting enhanced white matter integrity. These findings imply that production-related foreign language skills may have a specific impact on neural adaptations. Nonetheless, the mechanisms underlying skill-specific effects remain unclear, as few studies have examined this directly. Addressing this gap will require future research that systematically explore how individual skills contribute to structural plasticity.

By extending bilingualism research to long-term foreign language learners, our study highlights an important but often overlooked population. In many predominantly monolingual social societies, such as South Korea, learners acquire a foreign language primarily through formal education with little naturalistic exposure. Our findings show that meaningful neuroplastic changes are not exclusive to traditional immersive bilinguals. Instead, sustained learning under limited exposure can also reshape brain structures, thus broadening definitions of bilingualism and underscoring the need to include diverse learner profiles in neurocognitive research.

Moreover, our results suggest that long-term learning without immersion can still drive structural changes. Previous studies have reported gray matter volume increases after short-term language training (e.g., Mårtensson et al., 2012; Stein et al., 2012). Building on this evidence, we observed white matter enhancements and gray matter reductions in anterior brain regions, changes typically associated with later stages of neuroplasticity. We do not argue that immersive L2 exposure is unnecessary; rather, our study emphasizes that limited daily exposure combined with long-term learning experience in educational settings can also have a substantial impact on structural adaptations which is potentially comparable to those associated with more immersive experiences.

Our findings also carry implications for language education. Educational settings can provide structured opportunities for language learning and use to drive significant structural changes that support more efficient language control. Given that language control shares neural resources with domain-general executive functions, such educational experiences may also confer indirect cognitive advantages like those observed in bilingualism (Bialystok et al., 2012; Green and Abutalebi, 2013).

### 4.1 Limitations

Despite the novel contributions of this study, several limitations warrant consideration. First, since most participants acquired English primarily through formal education, the structural changes we observed could theoretically reflect general learning-related abilities rather than language-specific effects. However, we consider this possibility unlikely and suggest that the observed results are more plausibly attributed to language-specific experiences. Neuroimaging and lesion studies have consistently implicated brain regions such as the IPL in language control (Barbeau et al., 2017; Fridriksson et al., 2010; Sliwinska et al., 2015), and our findings of significant changes in these regions support a strong functional association with language-related demands. Moreover, prior studies that included non-language learning control groups provide further support for this interpretation. For example, Mårtensson et al. (2012) found significant tissue expansion in L2 learners compared with cognitive science students, suggesting that the structural adaptations specifically reflected language learning. Similarly, Schlegel et al. (2012) reported increased integrity in the genu of the CC only in L2 learners, not in GPA-matched controls. These findings support the notion that structural adaptations stem from language-specific experiences rather than general cognitive engagement. Additionally, the structural changes we identified in the present study correlated with daily foreign language use rather than proficiency, further suggesting that language experience drove these adaptations. Further research could strengthen this conclusion by including measures like general academic achievement as covariates.

Second, although our participants are more accurately described as long-term foreign language learners rather than bilinguals, variation in AoA could partly overlap with the “early” and “late” bilingual classifications used in previous research (e.g., Gracia-Tabuenca et al., 2024). We included AoA as a continuous predictor to account for this variability, but future studies with larger samples could further explore potential subgroup differences more systematically.

Third, since our participants acquired English in a non-naturalistic context, individual differences such as motivation, self-efficacy, and attitudes toward English may potentially have mediated or moderated the effects on neuroplasticity—factors we could not control. Future research should incorporate these variables to isolate the direct effects of language experience on neural adaptations.

Finally, while this study broadened the scope of previous work by focusing on an understudied population, our sample size was relatively small and lacked diversity in language proficiency and daily use experiences. Most participants displayed high proficiency but limited daily exposure. This homogeneity constrained our ability to explore more nuanced, experience-related neural changes, such as potential non-linear effects, and limited the generalizability of our findings. Future research should recruit more diverse groups of multilingual users and apply advanced statistical approaches, such as mixed models, to capture more complex relationships between language experience and brain structure (e.g., Korenar et al., 2023; DeLuca et al., 2024).

### 4.2 Conclusion

In conclusion, our study examined foreign language learners with limited daily exposure and investigated how their gray matter volume and white matter integrity related to AoA, proficiency, and daily use. Our results revealed that AoA and daily use predicted changes in gray matter volume and enhancements in white matter integrity in regions involved in language control and general executive functions. These findings suggest that even limited but long-term foreign language exposure can dynamically reshape brain structure. By extending neurocognitive models of bilingualism to include non-immersive learners, this study advances a more comprehensive understanding of experience-included brain plasticity.

## Data Availability

The raw data supporting the conclusions of this article will be made available by the authors, without undue reservation.

## Footnotes

1
http://www.fil.ion.ucl.ac.uk/spm/software/spm12

2
http://neuromorphometrics.com/

3
https://afni.nimh.nih.gov/
