# Supplementary material for: Brain structure correlates of foreign language learning experiences
Source: Front Hum Neurosci. 2025 Sep 18;19:1663218. doi: 10.3389/fnhum.2025.1663218 (PMC12488631; doi:10.3389/fnhum.2025.1663218)
Supplement: Supplementary file 1 [file Data_Sheet_1.pdf]

Supplementary Material

1 Supplementary Tables

Table 1. Significant and non-significant results from voxel-based morphometry analyses across cortical and subcortical regions of interest for language-related predictors.

Language-related factors	*Regions*	*Hemisphere*	*Direction*	*T*	*p_uncorr*	*x*	*y*	*z*
AoA	Angular cingulate cortex	Left	+	1.93	.032	-2	26	32
			-	0.82	.190	-2	42	-12
		Right	+	2.41	.012	9	36	-8
			-	0.21	.341	9	45	10
	Angular gyrus	Left	+	1.84	.038	-28	-69	56
			-	4.65	.000	-40	-74	34
		Right	+	3.03	.003	46	-50	57
			-	2.32	.014	62	-46	28
	Middle frontal gyrus	Left	+	1.89	.035	-39	45	0
			-	2.95	.003	-44	44	16
		Right	+	2.91	.004	44	57	-2
			-	1.77	.043	33	2	50
	Opercular part of the inferior frontal gyrus	Left	+	2.01	.027	-56	24	21
			-	1.25	.105	-54	12	-2
		Right	+	1.27	.102	54	15	30
			-	2.97	.003	56	16	0
	Orbital part of the inferior frontal gyrus	Left	+	0.97	.157	-51	26	-9
			-	3.36	.001	-42	30	-6
		Right	+	2.67	.007	50	44	-15
			-	1.55	.065	38	26	-2
	Triangular part of the inferior frontal gyrus	Left	+	2.48	.010	-56	32	12
			-	3.30	.001	-42	32	-4

		Right	+	1.35	.091	51	44	-9
			-	1.78	.043	42	44	-3
	Caudate	Left	+	1.53	.067	-18	18	3
			-	0.53	.260	-16	14	18
		Right	+	1.59	.061	18	22	-2
			-	1.26	.104	15	-14	21
	Putamen	Left	+	1.43	.079	-32	-9	6
			-	1.30	.097	-27	-12	-8
		Right	+	1.38	.086	18	18	-8
			-	0.99	.153	24	3	-10
	Thalamus	Left	+	1.00	.151	-12	-3	12
			-	4.22	.000	-15	-30	-6
		Right	+	1.23	.109	12	-3	12
			-	2.89	.004	10	-30	-3
Proficiency	Angular cingulate cortex	Left	+	1.82	.039	-2	24	30
			-	1.58	.062	-4	39	12
		Right	+	1.99	.029	2	24	30
			-	0.93	.165	0	22	-14
	Angular gyrus	Left	+	2.37	.013	-45	-68	48
			-	1.58	.061	-45	-76	34
		Right	+	2.73	.006	32	-64	48
			-	2.98	.003	40	-74	45
	Middle frontal gyrus	Left	+	2.10	.023	-24	40	24
			-	3.30	.001	-45	45	16
		Right	+	2.76	.005	30	24	46
			-	2.85	.004	28	-4	57
	Opercular part of the inferior frontal gyrus	Left	+	2.50	.010	-54	16	27
			-	1.11	.130	-45	20	8

		Right	+	1.76	.045	54	15	30
			-	1.97	.030	57	16	2
	Orbital part of the inferior frontal gyrus	Left	+	2.62	.007	-39	24	-9
			-	1.90	.034	-42	33	-8
		Right	+	3.01	.003	52	38	-10
			-	1.22	.110	38	24	-6
	Triangular part of the inferior frontal gyrus	Left	+	2.21	.018	-54	28	8
			-	1.77	.043	-44	33	-6
		Right	+	3.02	.003	52	40	-9
			-	1.19	.116	54	26	18
	Caudate	Left	+	2.08	.024	-12	-3	16
			-	0.65	.230	-16	18	14
		Right	+	1.62	.057	12	0	12
			-	1.75	.045	16	16	16
	Putamen	Left	+	1.13	.125	-28	-20	8
			-	2.06	.025	-26	-9	-8
		Right	+	1.04	.142	18	18	-8
			-	1.07	.137	32	-9	-12
	Thalamus	Left	+	1.77	.043	-6	-28	12
			-	2.62	.007	-24	-30	-3
		Right	+	1.55	.065	12	-3	14
			-	2.41	.012	20	-32	-4
Daily use	Angular cingulate cortex	Left	+	1.46	.075	-9	45	4
			-	3.36	.001	0	40	12
		Right	+	0.43	.284	0	22	-14
			-	3.69	.001	4	42	12
	Angular gyrus	Left	+	3.12	.002	-57	-57	24
			-	0.95	.161	-45	-64	14
		Right	+	4.22	.000	46	-64	36

		-	1.21	.112	46	-51	57
Middle frontal gyrus	Left	+	2.69	.006	-24	48	14
		-	2.35	.013	-27	62	0
	Right	+	2.11	.022	33	50	24
		-	2.15	.021	34	16	45
Opercular part of the inferior frontal gyrus	Left	+	0.48	.271	-44	21	20
		-	2.71	.006	-42	4	22
	Right	+	1.21	.112	58	26	18
		-	1.75	.046	54	15	27
Orbital part of the inferior frontal gyrus	Left	+	0.63	.236	-46	46	-10
		-	1.80	.041	-51	26	-9
	Right	+	0.92	.167	44	46	-8
		-	2.32	.014	45	26	-14
Triangular part of the inferior frontal gyrus	Left	+	1.73	.047	-39	40	6
		-	1.92	.033	-48	21	3
	Right	+	1.29	.099	42	40	0
		-	1.70	.049	40	32	16
Caudate	Left	+	0.64	.233	-15	16	-8
		-	1.81	.040	-16	10	20
	Right	+	1.71	.048	15	-14	20
		-	2.08	.024	15	9	21
Putamen	Left	+	2.26	.016	-26	-14	4
		-	-0.01	.395	-12	6	-9
	Right	+	2.39	.012	26	-9	8
		-	0.65	.230	33	-16	-4
Thalamus	Left	+	2.26	.016	-22	-24	9
		-	1.18	.118	-2	-3	3
	Right	+	2.64	.007	22	-21	9

				-	0.84	.184		2	-3	3

Note. Reported *p*_uncorr correspond to the uncorrected *p* value of the peak voxel in each region, and *x, y, z* coordinates indicate the peak voxel location in standard MNI space.

Table 2. Significant and non-significant results from tract-based spatial statistics analyses across tracts of interest for language-related predictors.

Language-related factors	*Tracts*	*Hemisphere*	*Measure*	*Direction*	*T*	*p_uncorr*	*p_FWE*	*x*	*y*	*z*
AoA	Corpus Callosum (genus)	-	FA	+	3.77	.022	.227	-7	29	9
			FA	-	3.05	.118	.757	5	28	6
			MD	+	3.75	.007	.136	7	28	8
			MD	-	2.88	.009	.644	-9	29	-3
	Corpus Callosum (body)	-	FA	+	3.33	.019	.499	-8	9	25
			FA	-	3.03	.002	.801	15	-12	33
			MD	+	3.88	.008	.261	10	-26	26
			MD	-	3.03	.019	.628	-15	-13	33
	Corpus Callosum (splenium)	-	FA	+	5.15	.001	.036	13	-36	23
			FA	-	3.42	.011	.592	16	-39	29
			MD	+	4.72	.000	.009	20	-46	13
			MD	-	3.47	.015	.664	-19	-52	22
	Superior longitudinal fasciculus	Left	FA	+	3.57	.013	.569	-32	3	20
			FA	-	3.21	.008	.286	-40	-44	16
			MD	+	3.29	.071	.882	-38	-43	16
			MD	-	3.94	.005	.299	-32	3	20
		Right	FA	+	3.20	.040	.697	35	-23	26
			FA	-	3.23	.026	.352	33	-36	35
			MD	+	2.69	.011	.620	35	-38	32
			MD	-	3.53	.015	.410	32	-30	37
	Inferior fronto-occipital fasciculus	Left	FA	+	3.50	.024	.308	-31	10	-6
			FA	-	1.64	.181	.922	-30	10	-9

			MD	+	2.15	.093	.802	-31	5	-9
			MD	-	3.27	.004	.521	-33	-10	-8
		Right	FA	+	3.41	.008	.095	35	-7	-12
			FA	-	3.58	.046	.452	35	-10	-8
			MD	+	2.32	.062	.792	28	15	-6
			MD	-	3.45	.001	.045	35	-8	-12
Proficiency	Corpus Callosum (genus)	-	FA	+	4.45	.010	.362	10	31	-2
			FA	-	3.99	.048	.627	-4	24	-2
			MD	+	2.77	.017	.777	7	28	8
			MD	-	4.38	.011	.137	12	29	12
	Corpus Callosum (body)	-	FA	+	3.25	.075	.721	5	3	25
			FA	-	2.61	.009	.921	-16	-21	32
			MD	+	3.49	.020	.583	8	11	24
			MD	-	3.26	.003	.745	15	-6	35
	Corpus Callosum (splenium)	-	FA	+	3.82	.013	.432	14	-37	24
			FA	-	3.67	.002	.235	24	-51	16
			MD	+	4.17	.006	.178	-12	-42	11
			MD	-	4.13	.015	.575	-20	-49	13
	Superior longitudinal fasciculus	Left	FA	+	2.68	.044	.825	-42	-46	8
			FA	-	2.97	.048	.528	-34	-25	27
			MD	+	3.90	.005	.280	-33	-37	34
			MD	-	3.26	.040	.875	-36	-24	34
		Right	FA	+	3.89	.024	.709	40	-42	15
			FA	-	3.63	.039	.530	36	-12	26
			MD	+	3.74	.001	.217	35	-29	27
			MD	-	2.94	.023	.294	34	-26	36
	Inferior fronto-occipital fasciculus	Left	FA	+	3.38	.011	.749	-32	-6	-4
			FA	-	3.19	.019	.154	-27	11	-9

			MD	+	4.08	.003	.069	-27	11	-10
			MD	-	3.38	.039	.792	-23	18	-9
		Right	FA	+	3.65	.026	.255	35	-1	-10
			FA	-	2.72	.083	.661	28	13	-7
			MD	+	1.97	.123	.992	33	4	-11
			MD	-	3.50	.003	.053	26	16	-9
Daily use	Corpus Callosum (genus)	-	FA	+	3.94	.010	.149	16	29	15
			FA	-	2.76	.002	.753	11	30	7
			MD	+	2.83	.004	.975	-2	27	2
			MD	-	4.44	.002	.040	13	21	21
	Corpus Callosum (body)	-	FA	+	4.18	.007	.266	17	1	35
			FA	-	4.28	.042	.906	10	-30	23
			MD	+	2.34	.043	.999	-15	-21	31
			MD	-	4.99	.000	.030	7	15	20
	Corpus Callosum (splenium)	-	FA	+	3.98	.006	.504	-20	-46	24
			FA	-	3.49	.004	.723	10	-31	23
			MD	+	3.35	.050	.961	10	-32	22
			MD	-	3.91	.001	.100	16	-43	23
	Superior longitudinal fasciculus	Left	FA	+	2.37	.079	.877	-40	-48	13
			FA	-	3.22	.013	.206	-34	-18	36
			MD	+	3.19	.037	.475	-38	-15	31
			MD	-	3.03	.027	.821	-35	-34	33
		Right	FA	+	3.64	.002	.241	36	-14	26
			FA	-	2.20	.127	.922	34	-17	36
			MD	+	2.24	.046	.950	35	-30	35
			MD	-	4.24	.004	.446	40	-48	16
	Inferior fronto-occipital fasciculus	Left	FA	+	3.24	.009	.112	-28	7	-9
			FA	-	2.78	.035	.536	-32	8	-5
			MD	+	3.62	.040	.542	-31	8	-7

	MD	-	2.99	.008	.196	-28	7	-9
Right	FA	+	3.76	.013	.184	34	-13	-5
	FA	-	1.74	.119	.833	34	6	-7
	MD	+	1.95	.127	.899	26	16	-8
	MD	-	3.99	.013	.390	34	4	-8

Note. *p*_uncorr correspond to the uncorrected *p* of the peak voxel in each region, and *p*_FWE correspond to the FWE-corrected *p* value of the peak voxel in each region. *x, y, z* coordinates indicate the peak voxel location in standard MNI space.
